# Biocompatible and Enzymatically Degradable Gels for 3D Cellular Encapsulation under Extreme Compressive Strain

**DOI:** 10.3390/gels7030101

**Published:** 2021-07-24

**Authors:** Zain Clapacs, Sydney Neal, David Schuftan, Xiaohong Tan, Huanzhu Jiang, Jingxuan Guo, Jai Rudra, Nathaniel Huebsch

**Affiliations:** 1 Department of Biomedical Engineering, McKelvey School of Engineering, Washington University in St. Louis, St. Louis, MO 63130, USA; zclapacs@wustl.edu (Z.C.); sneal@wustl.edu (S.N.); david.schuftan@wustl.edu (D.S.); xiaohong.tan@wustl.edu (X.T.); huanzhu.jiang@wustl.edu (H.J.); srudra22@wustl.edu (J.R.); 2 Department of Mechanical Engineering and Material Science, McKelvey School of Engineering, Washington University in St. Louis, St. Louis, MO 63130, USA; j.guo@wustl.edu

**Keywords:** hydrogel, alginate, GelMA, interpenetrating network, mechanosensing, scaffold

## Abstract

Cell encapsulating scaffolds are necessary for the study of cellular mechanosensing of cultured cells. However, conventional scaffolds used for loading cells in bulk generally fail at low compressive strain, while hydrogels designed for high toughness and strain resistance are generally unsuitable for cell encapsulation. Here we describe an alginate/gelatin methacryloyl interpenetrating network with multiple crosslinking modes that is robust to compressive strains greater than 70%, highly biocompatible, enzymatically degradable and able to effectively transfer strain to encapsulated cells. In future studies, this gel formula may allow researchers to probe cellular mechanosensing in bulk at levels of compressive strain previously difficult to investigate.

## 1. Introduction

Mechanical strains and stresses influence cell behavior and outcome in a wide variety of contexts [1,2,3,4,5,6]. For the study of cells under externally applied strain, there are two categories of techniques that can be used: those that apply strain to individual cells and those that apply strain to cells in bulk, within tissue or tissue-like materials [7]. In vivo, cells are typically loaded in bulk within tissue, where the loading affects the local osmotic environment and may alter paracrine signaling between cells [8].

For this reason, application of exogenous strain to engineered tissues or cells in hydrogels is often the most physiologically relevant way to study cellular mechanosensing pathways in vitro [7]. The primary methods for studying cell responses to mechanical loading in bulk are the application of loads to tissue explants and to cells seeded in scaffolds. Although compression of tissue explants allows cells to be loaded in their native milieu, this approach cannot be used for cells grown in vitro (e.g., cell lines) and limits researchers’ ability to study how the biochemical and biomechanical composition of the environment affect cellular responses to exogenous forces. Thus, the use of polymeric scaffolds represents an important approach for bulk cell loading in vitro [7].

A variety of materials have been used as scaffolds for cell encapsulation both for bulk compression of cells and for tissue engineering purposes, including polymer hydrogels [9,10,11,12,13,14]. Hydrogels are ideal scaffolds for their biocompatibility and ease of use, along with the ability to precisely engineer cell–ECM interactions by modifying the gel-forming polymers with adhesive proteins or peptides [15,16,17,18,19]. However, hydrogels that are compatible with encapsulating viable cells tend to withstand relatively low levels of compressive strain before failure. Even in tissue explants, strain levels in experimental studies rarely substantially exceed 20% [9,13,20,21]. This low strain level is in part due to the fact that common cytocompatible polymer hydrogels such as alginate and gelatin methacryloyl (GelMA), along with certain explanted soft tissues, typically fail at or below 50% compressive strain [22,23,24]. In contrast, existing hydrogels optimized for very high toughness and strain tolerance are frequently unsuitable for cell encapsulation due to toxic components or inhospitable gelation conditions [25,26,27]. This limitation presents a need for hydrogel scaffolds optimized for bulk compression of encapsulated cells at very high strain. An ideal gel for this purpose would not only withstand high levels of compressive strain without fracture, but also effectively transfer bulk compressive strain to encapsulated cells, promote favorable encapsulation conditions and withstand repeated high levels of compression. Additionally, to permit detailed analysis of the biologic changes resulting from compression, the ideal material would permit facile and quick recovery of encapsulated cells for further analyses (e.g., flow cytometry).

To develop such a gel, we investigated the use of both interpenetrating networks (IPNs) and multiple crosslinking mechanisms, two techniques known to increase hydrogel toughness [28,29]. We also investigated changes in polymer molecular weight to reduce dynamic viscosity of the sol to minimize shear forces experienced by cells during encapsulation [30]. To ensure facile recovery of encapsulated cells from the scaffold, we investigated the enzymatically degradable polymers, GelMA and alginate as matrix components. The resulting gel was able to maintain cell viability and transfer strain to encapsulated cells at extreme levels of compressive strain of at least 70%. Although GelMA and alginate IPNs are well known in the literature, to the authors’ knowledge they are yet to be simultaneously optimized for biocompatibility and high compression after cellular encapsulation. Rather, previous works investigating GelMA and alginate IPNs have demonstrated strains at failure of closer to 55% compression [31]. Similarly, previous investigations of GelMA and alginate IPNs have crosslinked the alginate and GelMA networks separately, with photocrosslinks not percolating between molecules of alginate and of GelMA [31,32,33]. This work demonstrates a GelMA and alginate IPN crosslinked by a novel method allowing photocrosslinks to percolate between the distinct matrix polymers and permitting compression to a regime previously difficult to access with biocompatible gels.

## 2. Results and Discussion

**Figure 1:**
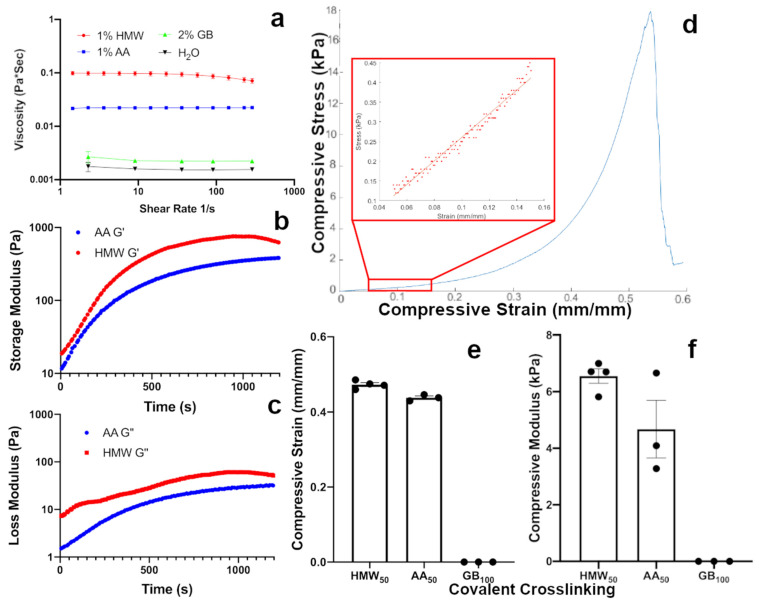
(**a**) The autoclaving and acid hydrolysis processes substantially reduced the dynamic viscosity of alginate solutions at shear rates ranging from 1 to 300 S^−1^. (**b**,**c**) AA and HMW alginate both form robust Ca^2+^ crosslinked hydrogels by internal gelation under time-sweep rheology conditions in under 20 min with plateauing storage (**b**) and loss (**c**) moduli. (**d**) Representative data of single uniaxial compression. (**e**) Compressive strain at failure of 1% (*w*/*v*) Eosin Y photocrosslinked AA_50_, HMW_50_, and GB_100_ molecular weights of methacrylated alginate. (**f**) Compressive modulus of 1% (*w*/*v*) Eosin Y photocrosslinked AA_50_, HMW_50_, and GB_100_ molecular weights of methacrylated alginate. Error bars represent standard error of the mean.

Alginate is biocompatible and easily crosslinked, making it an ideal material for cell encapsulation [34]. Alginate can be crosslinked both ionically through divalent cations and covalently, either through direct carbodiimide reactions, or through other chemistries if the polymer backbone is modified with reactive groups such as methacrylamide [34,35]. As carbodiimide reactions are toxic to cells, polymer backbone modification-based approaches are more suitable for cell encapsulation studies. Considering the advantages of an alginate network, we first investigated the potential to reduce shear forces experienced by cells during encapsulation in alginate gels by reducing the viscosity of the alginate sol. We investigated the sol viscosity and Ca^2+^ ionic gelation kinetics of three different types of alginate: (1) unmodified high molecular weight (HMW) with a molecular weight close to 250 kDa, (2) intermediate molecular weight polymers generated by autoclaving HMW alginate (AA) to a molecular weight close to 100 kDa, (3) and G-blocks isolated by acid hydrolysis of HMW alginate (GB) with a very low molecular weight close to 6 kDa [36,37]. Using shear flow cone viscometry at shear rates ranging from 1 to 300 S^−1^, we determined that all measured shear rates of AA have a dynamic viscosity close to half that of HMW, while the dynamic viscosity of GB was much lower, barely distinguishable from that of water (Figure 1a). This observation comports well with previous results suggesting similar relations in both synthetic and natural polymer sols [38,39].

We next assessed the crosslinking kinetics of these different alginate polymers in the presence of calcium. By chelating Ca^2+^ ions with ethylenediaminetetraacetic acid and dissolving in the sol we were able to internally gel the alginate on a parallel plate rheometer during a time-sweep experiment by pH-initiated unchelation of Ca^2+^ ions mediated by glucono–delta–lactone hydrolysis. In such experiments, both AA and HMW alginate gels achieved a plateau in storage and loss moduli in under 20 min and formed mechanically self-supporting hydrogels (Figure 1b,c). In contrast, solutions of pure GB failed to ionically crosslink at concentrations up to 10% weight per volume over a period of 2 h, and were, therefore, not investigated under time-sweep rheology. These studies confirmed that AA and HMW, but not GB, were sufficiently large to form a robust gel on their own through Ca^2+^ ion gelation.

After confirming that HMW and AA could form pure ionic networks, we investigated their abilities to form pure covalent networks. HMW and AA were modified with methacrylamide groups to a theoretical degree of substitution of 50% of alginate uronic acids and GB to a theoretical degree of substitution of 100% of alginate uronic acids, yielding HMW_50_, AA_50_ and GB_100_. Because photocrosslinking in a time-sweep experiment rheology study was unfeasible, gels were formed prior to testing by exposure to visible light in the presence of the Eosin Y photoinitiator system [40,41]. During testing, gels were subjected to single uniaxial compression to failure to generate a stress–strain plot (sample data Figure 1d). While both AA_50_ and HMW_50_ formed robust gels, GB_100_ was unable to form a self-supporting photocrosslinked gel (Figure 1e,f). This failure to gel may indicate that the molecular weight of GB is too low for bonds to percolate across the sol to form a single network. The strain at failure (Figure 1e) and compressive moduli (Figure 1f) of these covalent networks was within the range of materials typically used for bulk application of compressive strain to cells (e.g., agar gels [42]). Interestingly, despite differences in the shear modulus of ionically crosslinked AA and HMW gels (Figure 1b,c), there was no significant difference between either the compressive strains at failure or the compressive moduli of the covalently crosslinked AA_50_ and HMW_50_ gels. With confirmation that AA was able to form mechanically rigid gels both ionically and covalently, while reducing dynamic viscosity of pre-gel sol to enhance the viability of encapsulated cells. This formulation was used for all subsequent studies.

We next investigated several possible methods of incorporating multiple crosslinking modes and multiple polymer networks to improve gel toughness. To take advantage of multiple crosslinking modes, we combined the Ca^2+^ ion and Eosin mediated covalent crosslinking mechanisms investigated previously to form multiple network alginate hydrogels. We also investigated the use of interpenetrating networks composed of AA and GelMA or AA and GB. In particular, GelMA was chosen as a potential matrix material for the interpenetrating network because it is highly biocompatible and promotes cell adhesion by many types of somatic cells [43,44,45]. Specifically, to test these different modes of crosslinking we designed and investigated four different gel formulations detailed in Table 1.

**Figure 2:**
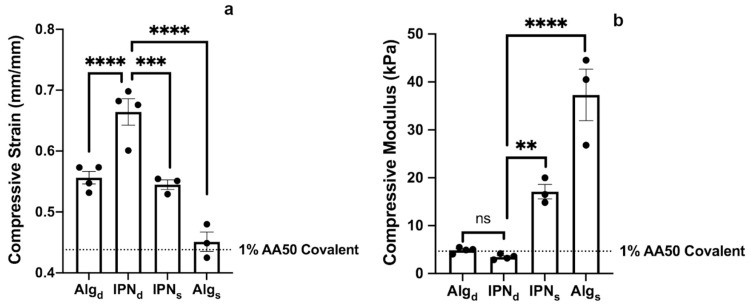
(**a**) IPN_d_ exhibited the greatest compressive strain at failure and Alg_d_ and IPN_s_ exhibited greater strain at failure than the pure alginate gels crosslinked with only Eosin. (**b**) The incorporation of the very low molecular weight GB_100_ alginate in Alg_s_ greatly increased the gel’s stiffness yielding the stiffest formulation tested, and the incorporation of GelMA without Ca^2+^ crosslinking in IPN_s_ increased the stiffness well above that of the 1% alginate covalently crosslinked gels. ** *p* < 0.01, *** *p* < 0.001, **** *p* < 0.0001 by one-way ANOVA and Tukey’s post-hoc multiple comparisons test. Error bars represent standard error of the mean.

Each formulation was tested similarly to the covalently photocrosslinked alginate hydrogels, to assess compressive strain at failure (Figure 2a) and compressive modulus (Figure 2b). By combining an interpenetrating network morphology with multiple crosslinking modes, IPN_d_ had a significantly greater compressive strain at failure than any other formulation tested. This result is consistent with previous work that observed interpenetrating networks with multiple crosslinking modalities tend to have high toughness and strain resistance [46]. Interestingly, while the incorporation of GelMA and two crosslinking modes increased strain at failure to the greatest extent (Figure 2a), the inclusion of methacryl modified GB in Alg_s_ increased the compressive modulus of gels the most (Figure 2b). Further, the formulations including calcium crosslinking (i.e., Alg_d_ and IPN_d_) were not substantially stiffer than the 1% alginate covalently crosslinked gels (Figure 2b). Moving forward we focused our efforts on IPN_d_, which demonstrated the greatest compressive strain at failure.

We expect that experimental studies on cellular mechanosensing may involve cell responses to cyclic strain in addition to single compressive events [32]. Thus, we tested the optimized gel formulation, IPN_d_, under cyclic loading. A similar setup to the one used for single compression was used, with the modification that instead of continuing compression until stopped manually, the experiment was set to compress the gel to a set strain, determined to be approximately 80% of the observed strain at failure (i.e., gels are compressed to 65% strain for IPN_d_, or 35% strain for 1% HMW_50_ with pure covalent crosslinks), and then decompressed to the zero point.

**Figure 3:**
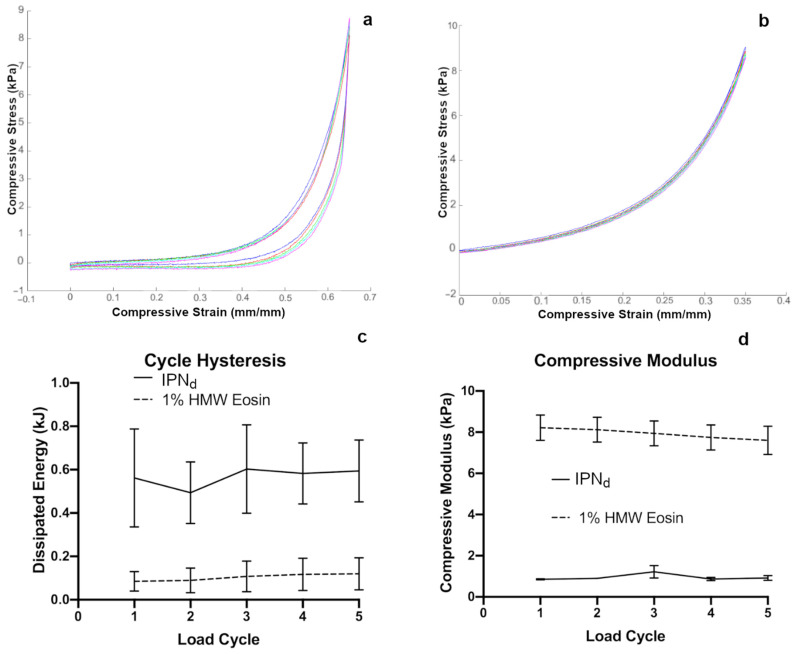
(**a**) Sample data for five loading cycles of IPN_d_ hydrogels to 65% compressive strain. (**b**) Sample data for five loading cycles of 1% HMW_50_ Eosin photocrosslinked hydrogels to 35% compressive strain. (**c**) Both the IPN and single network hydrogels showed no significant change in hysteresis over different loading cycles. (**d**) Both the IPN and single network hydrogels showed no significant change in compressive modulus over different loading cycles. Error bars represent standard error of the mean.

Gels in such compression were allowed to rest for no more than 10 s between compression cycles in order to simulate near-continuous cyclic compression, judged to be the most challenging mechanism of cyclic compression likely to cause fatigue failure [47]. Using similar procedures, the highly compressible IPN_d_ (sample data Figure 3a) was compared to more rigid 1% HMW_50_ Eosin crosslinked hydrogels (sample data Figure 3b). During compression, the compressive modulus was recorded similarly to single compression by approximating a zeroth order relationship between stress and strain between 5 and 15% compression during the compression cycle. The cycle hysteresis was calculated by using a nearest neighbor approximation for stress at a given point then computing the energy difference between the compression and decompression curves on each cycle. We found that for five consecutive cycles the compressive modulus and cycle hysteresis remained nearly constant with no significant deviation between the values recorded for a gel at different cycles (Figure 3c,d), however, the IPN_d_ and HMW_50_ networks differed substantially from one another. This is consistent with previous results investigating Alginate/GelMA IPN’s crosslinked by methods slightly different than those employed here, although the strain at failure of gels observed in this study is higher than in previous work [32].

**Figure 4:**
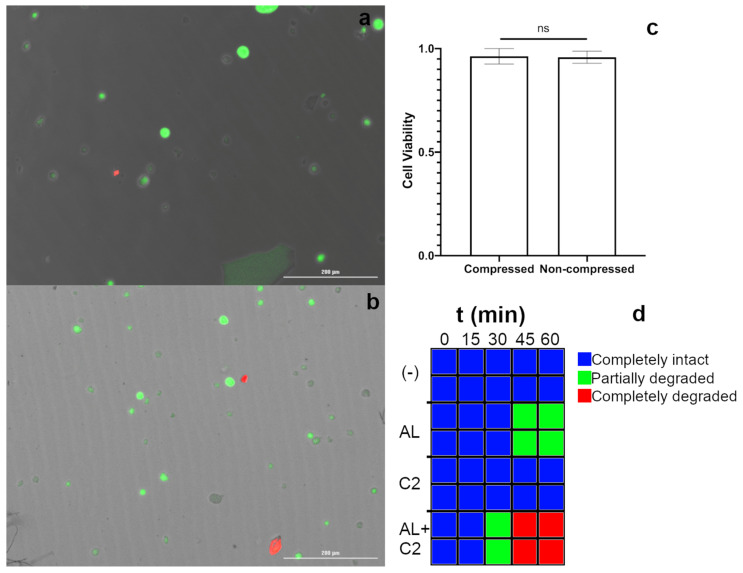
(**a**,**b**) Representative image of Calcein-AM and ethidium homodimer 1 stained D1 cells recovered from gels by enzymatic degradation three days post encapsulation, (**a**) two days post compression to 50% strain, (**b**) no compression. Live cells were stained green while dead cells were stained red. (**c**) Cells recovered from gels that had been compressed to 50% compressive strain appeared to have no significant difference in viability from cells recovered from uncompressed gels. (**d**) Degree of degradation of gels exposed to 1 mg/mL of alginate lyase, collagenase 2, both or neither at time points between 15 and 60 min. Gels were determined to be partially degraded if they were not self-supporting but remined visible, while gels, where no particulate or otherwise residue was visible, were determined to be completely degraded. Scale bars 200 μm. Error bars represent standard error of the mean. AL: alginate lyase; C2: collagenase 2.

With the mechanical properties of the gel confirmed to be suitable for studies involving high bulk compression, we next validated the cytocompatibility of these materials in cell encapsulation studies, and the ability to rapidly retrieve viable cells by enzymatically degrading the gels. We first investigated the degradation kinetics of IPN_d_ in solutions of alginate lyase, collagenase II, or both enzymes. Within a 1 h timeframe, 1 mg/mL concentrations of either alginate lyase or collagenase II alone weakened the gels but left the material largely intact. In contrast, the combination of both enzymes completely degraded IPN_d_ after only 45 min (Figure 4d).

With the feasibility of gel degradation confirmed, we next investigated the biocompatibility of the entire process of cell encapsulation, compression and enzymatic removal from gels. Because of the particular relevance of highly compressible scaffolds for cell encapsulation to bone and cartilage tissue engineering [5,48], we investigated the viability of D1 mesenchymal stromal cells (MSCs) [49], following the degradation of gels at day 3 post encapsulation. We first encapsulated cells in two groups of IPN_d_ hydrogels. Then 24 h after encapsulation, one group was subjected to a single cycle of 50% compressive strain under aseptic conditions and cultured for two more days to allow time for any compression triggered injuries to result in cell death. After this subsequent culture period, both compressed and non-compressed gels were enzymatically degraded to isolate cells, which were stained with Calcein-AM and ethidium homodimer 1 (live/dead stain; representative images Figure 4a,b). Both groups displayed viability well over 90%, and there was no significant difference observed between the viability of the cells in the compressed and uncompressed groups (Figure 4c). These results confirmed that cells could be recovered from IPN_d_ hydrogels by enzymatic degradation for further study. They also suggest that the process of encapsulation compression to moderate strain and recovery through enzymatic degradation may not have an appreciable impact on cell viability, although more substantial investigation may be merited to confirm this property.

**Figure 5:**
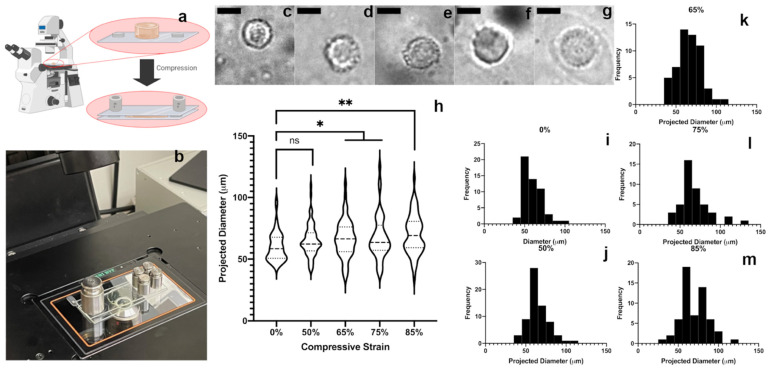
(**a**) A diagram of the process used for hydrogel imaging in compression. (**b**) A photograph of a hydrogel being compressed during imaging. (**c**–**g**) Representative images of in-focus cells at 0% (**c**), 50% (**d**), 65% (**e**), 75% (**f**) and 85% (**g**) compressive strain (gels failed at 85% compressive strain). Scale bars are 50 μm. (**h**) Violin plot of apparent cell diameter at 0, 50, 65, 75 and 85% compressive strain, with dashed line representing median and dotted lines representing first and third quartiles. Statistical comparisons refer to difference in sample mean from the 0% compression case, computed by one-way ANOVA with Holm–Sidak post-hoc analysis. (**i**–**m**) Histograms of projected cell diameter at 0, 50, 65, 75 and 85% compressive strain. Frequency is shown as absolute rather than relative frequency. * *p* < 0.0332, ** *p* < 0.0021. The number of cells imaged in each group varied between 45 and 65.

Finally, with the mechanical properties and biocompatibility of the gels confirmed, we investigated the potential of the gel to transmit bulk strain to encapsulated cells. D1 MSCs were encapsulated in IPN_d_ hydrogels and imaged in bright field under various levels of strain on a custom-built apparatus on an inverted objective microscope (Figure 5a,b). Initially, gels were imaged without any manipulation to obtain images of cells in uncompressed gels as a reference point. Next, the gels were sequentially compressed and imaged at 50, 65, 75 and 85% compressive strain (Figure 5c–g). Notably, gel fracture occurred between 75 and 85% compressive strain. At higher compression levels, the typical observed cell diameter increased significantly from that observed in non-compressed gels (Figure 5h–m). If MSCs cultured in a 3D scaffold are assumed to be approximately spherical, an assumption supported by previous work and images obtained in this study [50], then an increase in observed cell diameter in response to compression can be reasonably interpreted as the result of encapsulated cells adopting an oblate spheroid morphology in response to compressive loading, an effect that has been observed in previous studies of somatic cells [20]. With this assumption in mind, it appears that the designed scaffold is successfully able to transfer bulk strain to encapsulated MSCs, making it a valuable tool for studying cells at levels of compressive strain previously difficult to probe. Because the scaffold appeared to transfer strain to encapsulated MSCs, this provides strong evidence of adhesion of MSCs to the scaffold, a result which is not particularly surprising because of previous work demonstrating that GelMA allows a wide variety of cell types to adhere tightly [43,44,45]. Indeed the material composition of the scaffold lends itself well to promoting cellular adhesion and tuning cell–matrix interactions through the modification of alginate with various syndecan- and integrin-binding peptides including the RGD motif [34,51,52,53]. This ability to easily modify alginate to differing degrees with various cell-interactive peptides allows for the modification of IPN_d_ to accommodate a wider variety of cell types than might be possible with a polymer that is less readily matrix-functionalized. Interestingly, along with the observed increase in apparent cell diameter as compression increases, the distribution of cell diameters at higher levels of compressive strain appears to be much wider at higher levels of compression, with the presence of a low-cell diameter tail that is less apparent at lower strain conditions. It is possible that this corresponds to cell contraction in response to strain-induced injury, a phenomenon that has been previously observed in cell types other than those studied here, including neuronal cells [54]. Although such an effect has not been previously reported in D1 MSCs, it is possible that a similar effect may be responsible for the increased prevalence of low apparent diameter cells at a higher compressive strain. This, combined with cellular deformation into an oblate morphology in response to mechanical loading may account for the wider observed distribution of cell diameter at greater compression levels. With this interpretation in mind, it is possible to infer that at greater than 65% compressive strain, MSC viability is severely compromised.

**Figure 6:**
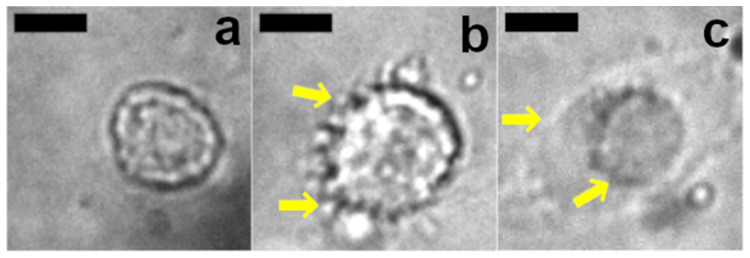
Representative images of three primary phenotypes observed during compression. (**a**) A typical healthy appearing cell. (**b**) A cell in the process of blebbing, note the surrounding detached bodies indicated by yellow arrows. (**c**) A cell displaying a phenotype which may correspond to compression injury, note the visible corona and inner cell body indicated by yellow arrows. Scale bars are 50 μm.

Interestingly, cells under compression exhibited three distinct phenotypes. At most compression levels, the majority of cells adopted a “healthy” phenotype consisting of a rounded morphology without any other particularly distinctive features (Figure 6a). At all compression levels, a substantial minority of cells appeared to be blebbing (Figure 6b). Interestingly, although a distinct trend toward increased projected cell diameter as a function of the level of applied compressive strain was observed (Figure 5h), the “blebbing” population appeared at roughly the same frequency (approximately 20% of cells) at all compression levels. The most unusual phenotype, cells with a visible corona (Figure 6c), was only observed at higher compression levels, and at 85% compressive strain, it became the dominant phenotype observed. This population represented close to one third of cells at 65 and 75% compressive strain, and at 85% compressive strain represented close to half of all observed cells. This phenotype likely corresponds to cells with compression-induced injury that resulted in membrane rupture or damage associated with osmotic change which has led to somewhat similar appearing phenotypes in previous work [55], although further study would be required to confirm that this is indeed the case.

The results of this study suggest several promising lines of future inquiry to confirm and expand upon the observed properties of the designed gel. Because IPN_d_ is intended for use at very high compressive strain there is a considerable likelihood that it is intended to operate outside of the linear viscoelastic regime. Rheological investigation determining the extent of linear viscoelasticity and the behavior of the gel at strains outside that range may prove valuable in applications of the hydrogel to investigate cellular response to very high bulk strain. Similarly, frequency-sweep rheological determination of the IPN_d_’s mechanical spectrum would allow for greater understanding of the gel’s behavior at different loading rates and determination of the swollen gel’s Poisson’s Ratio, which can be used to relate the shear and compressive moduli. Valuable future lines of study would also include further investigation of IPN_d_’s biological interactions. For example, maintenance not only of overall cellular metabolic activity but also phenotype, as addressed by the transcriptome and protein expression analysis, would provide a more robust illustration of this materials’ influence on the biology of encapsulated cells. Importantly, the degree to which strain can be transferred from a scaffold to encapsulated cells is largely dependent on the degree and mechanism through which cells adhere to the scaffold material. Future investigation on the degree to which various somatic cell lines and primary cells adhere to the hydrogel matrix will shed light on the applicability of the scaffold to different cell types and the ability to transfer mechanical loads to cells other than D1 MSCs.

## 3. Conclusions

The gel designed in this work demonstrated several properties making it uniquely favorable for the study of encapsulated cells at very high compressive strain by employing a novel crosslinking method to achieve compressive strain tolerance that improves upon previous designs. The gel was designed to have a reduced viscosity sol to accommodate shear-sensitive cells, full enzymatic degradability on a laboratory-friendly time scale to facilitate the analysis of encapsulated cells, high biocompatibility of encapsulation and compressive strain tolerance above 70% with rapid recovery and the ability to transfer bulk strain to encapsulated cells. The gel presents the opportunity for researchers to study cellular mechanosensing at levels of strain previously difficult to investigate and may prove invaluable to future tissue engineering and mechanobiology research.

## 4. Materials and Methods

### 4.1. High Molecular Weight Alginate 50-Methacrylate (HMW_50_) Preparation

**Figure 7:**
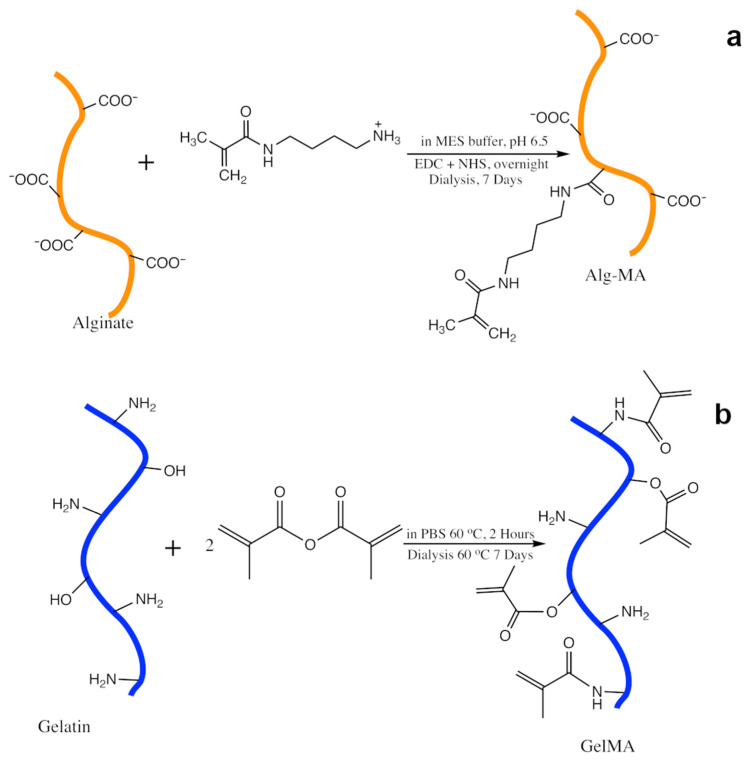
(**a**) Schematic of Alginate–MA preparation (**b**) Schematic of GelMA preparation.

HMW_50_ was prepared following similar methods to those used previously by Jeon et al. [56]. MES buffer was prepared by dissolving 1.952 g of MES (2-(N-morpholino)ethanesulfonic acid) and 1.75 g of NaCl into 100 mL of DI H_2_O and adjusting the pH to 6.5 with HCl and NaOH. 1 g of GMB Manugel (FMC biopolymer, Philadelphia, PA, USA) was then dissolved in the buffer. Once the alginate was dissolved, 173.6 mg of N(3-aminopropyl)methacrylamide (Sigma-Aldrich, St. Louis, MO, USA), 554.6 mg of EDC (1-Ethyl-3-(3-dimethylaminopropyl)carbodiimide) (Chem-impex, Wood Dale, IL, USA (Chem-Impex)) and 166.4 mg of NHS (N-hydroxysuccinate)(Chem-Impex) were added and the solution was left to react overnight (Figure 7a). Once reacted, the solution was dialyzed in 10kD-cutoff dialysis tubing against a NaCl gradient in DI H_2_O starting at 7.5 g/L, which was reduced by 1.25 g/L every time the dialysis solution was changed approximately every 3 h. The dialysate was then frozen and lyophilized.

### 4.2. Autoclaved Alginate 50-Methacrylate (AA_50_) Preparation

GMB Manugel was dissolved in deionized water at a concentration of 1% weight per volume, and autoclaved in wide mouth glass bottles with low pressure steam for 10 min at 121 °C. This is a process that has been previously observed to substantially reduce the molecular weight of alginate [57]. Next the resulting solution was dialyzed in 10 kD-cutoff dialysis tubing against a NaCl gradient in deionized water (DI H_2_O) starting at 7.5 g/L, which was reduced by 1.25 g/L every time the dialysis solution was changed approximately every 3 h. The dialyzed solution was frozen and lyophilized until dry. Once dry, methacryl groups were conjugated to the polymer using the same methods as above for the production of HMW_50_ and the polymer was then dialyzed, frozen and lyophilized.

### 4.3. G-Block Alginate 100-Methacrylate (GB_100_) Preparation

GB_100_ represented the lowest molecular weight variant of alginate used in creating gels for this project, it is created by lysing higher molecular weight variants of alginate and isolating only the guluronic acid blocks of the heavier alginate chain by acid hydrolysis as described previously by Bouhadir et al. [37]. Specifically, the procedure followed was as such: GMB Manugel was suspended in DI H_2_O at 2% *w*/*v* until fully dissolved then heated to 90 °C. 5.6 mL of 3 M HCl solution was added dropwise per gram of alginate, then the solution was covered and allowed to mix at 90 °C for 4 h. After 4 h, the solution was vacuum filtered, and the solid phase was reserved and resuspended in DI H_2_O at 1.4 times the original volume along with 390 mg of NaCl and 14.3 uL of 4 M NaOH for each mL of the original 2% solution. Once fully dissolved the alginate was reprecipitated by adding 267 uL of 12 M HCl per g of alginate originally added. The solution was filtered again, then resuspended and precipitated again as described above once more. After that second precipitation, the solution was once again filtered, however, this time the solid phase was resuspended in DI H_2_O to 0.7 times the original volume of the original 2% solution. The pH was then adjusted to 7.5 using 4 M NaOH, and the solution was decolored by adding 266 mg per original g of alginate of fine mesh activated charcoal. The charcoal was mixed then allowed to settle for 2–16 h, then the solution was filtered through a 0.2 to 0.4 um mesh. The filtrate was reserved, then from the filtrate the alginate was precipitated with the addition of 2.1 L of ethanol per L of the original 2% solution. The precipitated polymer was again vacuum filtered, then resuspended in 0.525 L of DI H_2_O per L of the original solution then once again precipitated with ethanol, this time with 1.575 L of ethanol per L of original solution. The polymer was once again vacuum filtered out and then dried in a desiccator vacuum chamber. Once fully dried, the polymer was resuspended at 50 mg/mL in DI H_2_O, frozen and lyophilized. The powder was then methacrylated using the same protocol as previously, increasing the concentrations of methacrylate, NHS and EDC two-fold.

### 4.4. Gelatin Methacryloyl Preparation

Gelatin methacryloyl (GelMA) was created using methods previously described in the literature by Kerscher et al. [40]. Briefly, Gelatin Type B-Bovine (Sigma) was suspended at 5% in PBS at 60 °C and allowed to react with methacrylic anhydride suspended at 15% for 2 h, then stopped by dilution with PBS (Figure 7b). The solution was then dialyzed against DI H_2_O for 1 week, changing the dialysis bath approximately daily. The dialysate was finally frozen and lyophilized.

### 4.5. Hydrogel Preparation

Hydrogels for mechanical testing were created in batches of 1.4 mL, equivalent to approximately 4 individual gels of 350 uL. First, the appropriate polymer or polymers (GelMA, AA_50_, GB_100_, or HMW_50_) were dissolved in an appropriate volume of PBS to ensure the proper final concentration in the gel.

If the gel was to be crosslinked with calcium ions, a solution of 400 mg/mL glucono–delta–lactone (GDL) (Sigma) was prepared separately as well as a 0.165 M Ca-EDTA solution made by dissolving both EDTA and any source of Calcium ions, in our case, CaCl_2_ salt at a concentration of 0.165 M in DI H_2_O and carefully adjusting the pH to 7.2 using 10 M NaOH and 10 M HCl. To initiate ionic crosslinking 233.4 uL of 0.165 M, the Ca-EDTA solution was added to the polymer suspension along with 466.8 uL of the prepared GDL solution, to a total volume of 1400 uL.

If the gel was to be covalently crosslinked at the methacryl groups, 105 uL of 20% triethanolamine in PBS was added to the solution along with 4.76 uL of N-vinylpyrrolidone (Sigma) and 4.62 uL of 1.5 mM Eosin Y (Sigma) in EtOH. If both methods of crosslinking were to be used, then both groups of chemicals were added. In all cases the final volume was 1400 uL. Once the complete sol was prepared, 4 gels were plated at 350 uL on a clear acrylic plate, then a second acrylic plate was laid over the top of the prepared gels at a height of 2 mm. To initiate covalent crosslinking, the gels were exposed to bright visible spectrum light for a minimum of 5 min; to initiate calcium crosslinking, the gels were left undisturbed for 2 h to allow the GDL solution to slowly acidify the gel and the EDTA to release the Ca^2+^ ions into the gel. The same procedure is used for the encapsulation of cells into hydrogels with modifications described in Section 4.9.

### 4.6. Hydrogel Compression Testing

The stiffness and toughness of the hydrogels were tested using an Instron model 5583 compression tester (Instron, Norwood, MA, USA). The gels were placed on top of the lower platen while the upper plan as lowered on top of the gel during compression at a rate of 1 mm/min, while force and extension were recorded with a 500 N load cell. These values were then used to calculate compressive stress and strain which were then used to calculate compressive modulus, stress and strain at failure and toughness with MATLAB (Mathworks, Natick, MA, USA ). Each load cycle was used to generate a stress–strain plot similar to the sample data shown in Figure 1d. The point of failure was interpreted to be the point at which stress was greatest, and each curve was manually inspected to ensure this assumption was accurate. The compressive modulus was estimated using a zeroth order fit of data between 5 and 15% compressive strain to remain within the linear compression region and avoid effects stemming from the low compression toe-region.

### 4.7. Hydrogel Degradation

Hydrogels for degradation, both for observation and for removal of encapsulated cells, were submerged in a solution of 1 mg/mL alginate lyase (Sigma) and 1 mg/mL collagenase 2 (ThermoFisher, Waltham, MA, USA) in PBS. The gels were then placed in an incubator at 37 °C, and occasionally removed to gently swirl at 5 min increments. Gels studied for the rate of degradation rate were removed from solution and their level of degradation was observed. Gels were observed every 15 min and if, at those times, they were mechanically rigid enough to be weighed, their wet weight was recorded, otherwise they were categorized as either partially degraded for gels that were still visibly present to some extent or completely degraded where no gel was visible at all.

### 4.8. Live/Dead Cell Staining

Cells were stained either in a gel or in 2D culture after degradation with Calcein-AM and ethidium homodimer 1 as live and dead cell dyes, respectively, (Live/Dead staining kit; ThermoFisher) following the manufacturer’s instructions.

### 4.9. Cell Encapsulation

Gels for cell encapsulation were prepared with the same formula as the gels described in Section 4.5 with the modification that the solvent used, instead of PBS, was serum-free high glucose DMEM without phenol red. To encapsulate, the cells were first pelleted then resuspended in the sol to a final concentration of 10^6^ cells/mL. Once the cells were resuspended the sol was gelled similarly to gels for compression testing. Once formed, gels were transferred to a bath of high glucose DMEM supplemented with 10% FBS.

### 4.10. Microscopy

Fluorescently stained cells were imaged using a BioTek Lionheart FX automated microscope (BioTek, Winooski, VT, USA). Bright Field Images of cells in compression were obtained using a Nikon Eclipse Ts2R inverted microscope. To image hydrogels in compression, cells were encapsulated in hydrogels as described previously in Section 4.9, then those hydrogels were immediately collected and placed in a bath of high glucose DMEM. Once a gel was to be imaged it was transferred to a microscope slide and placed on the stage of the Nikon microscope. For uncompressed imaging, the gels were first imaged as typical without compression. To compress the gels, several 0.17 mm coverslips were placed on either side of the gel on the microscope slide (Figure 5a,b). The number was selected so that their combined thickness was approximately 1 mm for 50% compression. To compress, a second microscope slide was placed on top of the gel and several 10 g weights on either side to compress the gel to the thickness of the stack of coverslips. For higher compression levels the weights and top slide were removed, and the number of coverslips was changed in each stack to a thickness appropriate for the level of compression. Then the slide and weights were added again to recompress the gel. At each compression level several images were taken before proceeding to the next higher level of compression. After image collection the in-focus cells from each image were analyzed using FIJI image processing software (Ver. May 2021, Open Source). During analysis, each cell’s apparent diameter was measured and phenotype noted.

### 4.11. Cell Culture

D1 mesenchymal stromal cells were used for all experiments. Cells were cultured in high-glucose Dulbecco’s Modified Eagle’s Medium (DMEM) with 10% Fetal Bovine Serum, 1% penicillin/streptomycin and 1 mM sodium pyruvate. Cells were used before passage 5.

## Figures and Tables

**Table 1:** Components and crosslinking mechanisms of investigated gel formulations.

Name	Matrix Material(s)	Crosslinking Method(s)
Alg_d_	1% AA_50_	Ca^2+^ ion + Photocrosslinking
IPN_d_	1% AA_50_ 4% GelMA	Ca^2+^ ion + Photocrosslinking
IPN_s_	1% AA_50_ 4% GelMA	Photocrosslinking
Alg_s_	1% AA_50_ 4% GB_100_	Photocrosslinking

## Data Availability

The data presented in this study are available in the article.
